# Abnormal peripheral chemokine profile in Huntington’s disease

**DOI:** 10.1371/currents.RRN1231

**Published:** 2011-04-13

**Authors:** Edward Wild, Anna Magnusson, Nayana Lahiri, Ulrika Krus, Michael Orth, Sarah J Tabrizi, Maria Björkqvist

**Affiliations:** ^*^UCL Institute of Neurology, London, UK; Bristol University, UK; ^†^Department of Experimental Medical Science, Wallenberg Neuroscience Center, Lund University, Lund, Sweden; ^‡^Department of Neurodegenerative Disease, UCL Institute of Neurology, London, UK; ^§^Lund University Diabetes Center Malmö Sweden and ^¶^Department of Neurology, University of Ulm, Ulm, Germany

## Abstract

Huntington’s disease (HD) is an inherited neurodegenerative disorder characterized by both neurological and systemic abnormalities. Immune activation is a well-established feature of the HD brain and we have previously demonstrated a widespread, progressive innate immune response detectable in plasma throughout the course of HD. In the present work we used multiplex ELISA to quantify levels of chemokines in plasma from controls and subjects at different stages of HD. We found an altered chemokine profile tracking with disease progression, with significant elevations of five chemokines (eotaxin-3, MIP-1β, eotaxin, MCP-1 and MCP-4) while three (eotaxin-3, MIP-1β and eotaxin) showed significant linear increases across advancing disease stages. We validated our results in a separate sample cohort including subjects at different stages of HD. Here we saw that chemokine levels (MCP-1 and eotaxin) correlated with clinical scores. We conclude that, like cytokines, chemokines may be linked to the pathogenesis of HD, and that immune molecules may be valuable in tracking and exploring the pathogenesis of HD.

## Introduction

Huntington’s disease (HD) is an inherited neurodegenerative disease, caused by a CAG triplet repeat expansion in the gene encoding huntingtin, for which there is no effective disease-slowing treatment. Many clinical features of HD can be ascribed to the dysfunction and death of neurons but evidence is emerging of a role for non-neuronal cells and tissue in the pathogenesis of HD. The causative mutant huntingtin protein is expressed ubiquitously [1], and several clinical disease features cannot be accounted for by neuronal pathology alone [2]. In neurodegenerative diseases in general, non-neuronal tissue and cells (such as astrocytes and microglia) are increasingly thought to influence neuronal dysfunction and death [3] [4].

The immune system has parallel representations in peripheral and central nervous system tissues. There is strong evidence of activation of microglia in areas of neuronal loss in HD brains, even before the emergence of clinical features, as well as in manifest disease and post-mortem [5] [6] We recently showed that activation of the innate immune system - likely induced by mutant huntingtin acting directly within inflammatory cells - occurs both peripherally and centrally throughout the course of the disease. Cytokines suggestive of an innate immune response were more abundant in HD plasma, even many years prior to predicted disease onset, and were linked to clinical progression [7]. This echoes the findings of others that immune system pathways may be of importance for our understanding of the pathogenesis of HD [8] [9]and a possible source of disease-slowing treatments [9].  

The promise of forthcoming clinical trials for HD-modifying therapies increases the necessity for objective markers of progression (‘state biomarkers’) in HD. The disease advances slowly, with a course that varies considerably between patients, Moreover, though progress has been made towards developing panels of biomarkers [10], there are no validated methods to assess underlying disease progression in gene carriers who have no overt disease signs [11]. Reliably quantified plasma markers that track with disease progression would be an asset in conducting clinical trials in HD.

The chemokine system is a large family of small molecules and twenty receptors, related to, butdistinct from cytokines, and having common roles as leukocyte chemoattractants. Chemokines are recognised as central to many processes related to infection and immunity, including migration of leukocytes into the CNS and modulation of the function of the blood-brain barrier [12]. Quantification of chemokine levels may shed light on the status of both the immune system as a whole and the function of specific immune components in HD. Chemokines have been implicated in the pathogenesis of other neurodegenerative diseases [4] and interesting recent results link the expression of mutant huntingtin in neurons to increased expression of chemokines [13].  

We quantified chemokine levels in plasma from HD patients and control subjects and evaluated their ability to distinguish different clinical stages. We validated our results in a separate sample cohort including subjects at different stages of HD and investigated whether chemokine levels correlated with clinical scores.

## **Material and Methods**

**Collection and processing of human plasma samples.** The study was conducted in accordance with the declaration of Helsinki and was approved by local ethics review boards; all subjects gave informed written consent. Blood samples were obtained from control subjects and genetically-diagnosed HD patients and processed as previously described [14]. Clinical assessment was carried out by a neurologist experienced in assessment of HD patients. Samples from cohort 1 were collected in London, UK and samples from cohort 2 were collected in Ulm, Germany. Subjects’ demographic data are given in Table 1.

**Plasma analyses. ** Plasmachemokine levels (Eotaxin, Eotaxin-3, IP-10, MCP-1, MCP-4, MDC, MIP-1b, TARC) were quantified using Meso Scale Discovery (MSD®, Gaithersburg, MD) electrochemoluminescence assays using a modification of the manufacturer’s protocol. 30 ul was used as the sample volume and a 10-point standard curve was used, ranging from 2500 pg/ml to 0 pg/ml. The sample and calibrator were incubated on the MSD plate for 3 h (instead of 2 h), followed by a wash (as per manufacturer’s recommendation). The MSD plate was then incubated with detection antibody solution for 3 h (instead of 2h) before wash and read as per manufacturer’s recommendation).  Results were analyzed on a SECTOR™ 6000 instrument (MSD). The operator was unaware of the disease state of each sample during processing and statistical analysis was performed independently. 

**Statistical analysis. **For the human plasma chemokine data, inter-group differences were identified by one-way ANOVA with post-hoc Tukey HSD testing to allow for multiple comparisons. As previously described, linear regression analysis using coded variables for each subject group (control=1, premanifest=2, early=3, moderate=4), using age and sex as covariates, was used to identify significant change with advancing disease [14]. Correlations with clinical variables were examined using linear regression analysis using sex as a covariate.

## **Results**

**Table 1. ** Demographic characteristics of subject cohorts

**Table:** 

Disease stage	Number of subjects	Female:male	Mean age (range)	Mean CAG (range)
**Cohort 1**				
Control Premanifest Early Moderate	34 15 23 27	22:12 8:7 11:12 19:8	44 (25-65) 39 (23-54) 47 (31-65) 52 (26-76)	- 42 (39-45) 44 (41-51) 45 (41-55)
**Cohort 2**				
Premanifest Early Moderate Advanced	26 44 16 8	13:13 31:13 9:7 4:4	42 (27-62) 52 (30-75) 51 (34-66) 63 (40-78)	43 (40-50) 44 (40-53) 46 (40-52) 44 (40-50)

**Figure 1.**
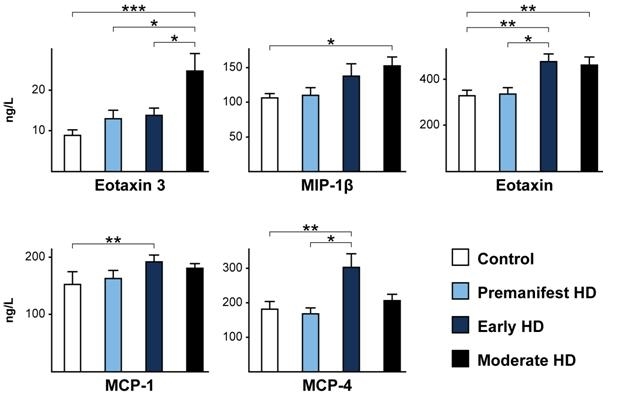
Plasma levels of individual chemokines by clinical group.

We collected two separate cohorts of plasma samples; cohort 1, 99 plasma samples from HD mutation carriers ranging from premanifest to moderate HD and from control subjects and cohort 2, 94 plasma samples from HD mutation carriers ranging from premanifest to advanced HD (Table 1) and quantified levels of key chemokines using multiplex sandwich ELISAs. In samples cohort 1, we found an altered profile of chemokine levels in HD patients. As seen in Figure 1, there were statistically significant elevations from control levels for five chemokines, at one or more disease stages. Additionally, statistically significant differences in chemokine levels were found between HD clinical stages for eotaxin-3, eotaxin and MCP-4.

*p<0.05;

**p<0.01; ***p<0.001 by Tukey HSD test (adjusted for multiple comparisons).

Three chemokines (eotaxin-3, MIP-1β and eotaxin) showed statistically significant increases across all subject groups with advancing disease (Figure 2).  

**Figure:**
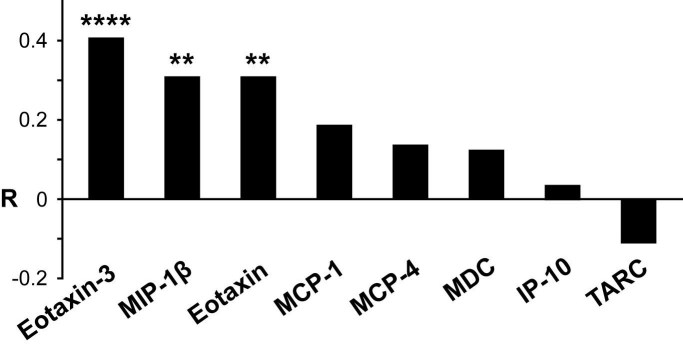
Figure 2. Overall relationships between plasma chemokine levels and disease stage. Increases with advancing clinical stage, analyzed using linear regression, were statistically significant for eotaxin-3, MIP-1β and eotaxin.  R-values (partial correlation coefficients) are corrected for age and sex. **p<0.01; ****p<0.0001.

Following our findings in sample cohort one, that several chemokines alter with disease stages, we evaluated a separate sample cohort, including 94 plasma samples from HD mutation carriers ranging from premanifest to advanced HD (Table 1). MCP-1 and eotaxin were significantly associated with UHDRS motor score scores as well as with decreasing function scores (Figure 3).

**Figure:**
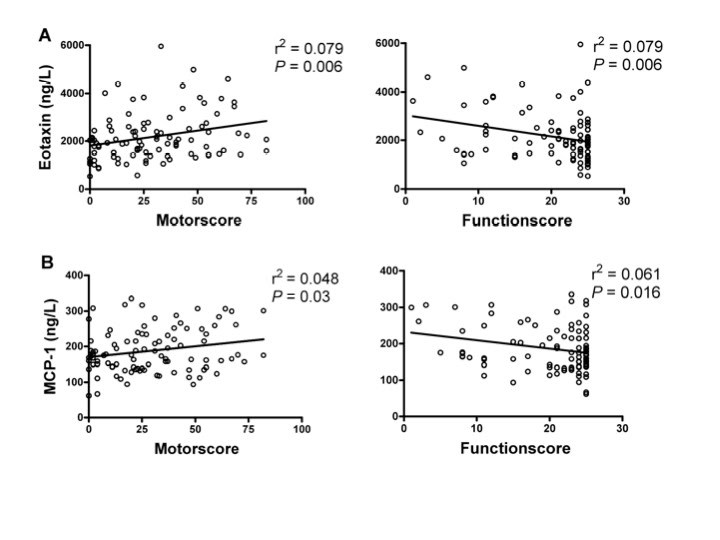
Figure 3. Correlations between plasma eotaxin levels (A) and MCP-1 (B) and clinical severity scores in premanifest and manifest HD gene carriers. Levels of eotaxin and MCP-1 correlated with worsening disease as demonstrated by increasing UHDRS motor scores and decreasing total function score.

## **Discussion**

Immune dysfunction is recognized in many neurodegenerative diseases, and increasingly a direct role in disease progression has been suggested [3] [4]. Chemokines have been implicated in other neurodegenerative diseases [4] [15]. For example, cerebrospinal fluid levels of IL-8, IP10 and MCP-1 [16], and plasma levels of eotaxin [17] and MCP-1 [18], were independently shown to be increased in Alzheimer’s disease and recently IP10 and eotaxin was suggested markers for age-related macular degeneration [19]. Also, plasma chemokine levels have been shown to correlate with disease progression in Parkinson’s disease [20]. In HD, our previous results suggest that inflammatory changes detected in peripheral plasma may be biologically relevant and mirror the neurodegenerative process occurring in the CNS [7].

Here we have shown that plasma levels of the chemokines eotaxin-3, MIP-1β, eotaxin, MCP-1 and MCP-4 are statistically significantly elevated above control levels in HD, with the first three tracking significantly with disease progression.  In a separate sample cohort we showed that chemokine levels (MCP-1 and eotaxin) correlated with clinical scores - positively with UHDRS motor scores and negatively with function scores. These results extend the potential scope of the interactions between the immune system and the pathogenesis of HD. 

Interestingly, it was recently shown that expression of mutant Huntingtin in a neuronal cell line increased expression of the chemokines MCP-1 and KC [13]. The NFkB/IKK signaling pathway has been proposed as a nexus of interaction between HD and the innate immune system. Activation of the chemokine system strengthens this hypothesis [21] and additionally implicates proteasomal dysfunction [13] and the Jak/STAT pathways [22] in HD immune dysregulation.

Taken together with previous results, these findings support the hypothesis that toxic effects of mutant huntingtin in immune cells that might contribute to pathogenesis in HD. It is, however, still uncertain how specific chemokine alterations might influence HD pathology. Further work is needed to explore what cells are responsible for the increase in circulating chemokines. Also, future work is needed in order to show how immune alterations could potentially be modifiers of HD progression. It would be very interesting to see how bone marrow transplantation or other immune-altering strategies in mouse models of HD, could influence pathogenesis in HD.

It will likely be challenging to use immune markers as biomarkers of disease progression or diagnostic predictors. Levels of many immune molecules are altered by infection and concomitant inflammatory illness.  Cytokines also display diurnal variation [23] and other factors likely influence plasma cytokine levels. Also, there might be technical limitations in assaying low abundance cytokines. The sensitive, specific genetic test for HD may obviate some of these challenges and several chemokines are present in plasma at higher concentrations than for example cytokines, increasing the possibilities of detecting robust changes across disease stages.

We conclude that the chemokine system and related immune pathways warrant further study in relation to the pathogenesis of HD, and among potential candidate biomarkers in future large-scale longitudinal studies.    

## **Funding information**

This study was financially supported by the CHDI Foundation, New York, and also funded in part by the UK Department of Health, the Medical Research Council (UK), the Wellcome Trust, BenteRexed foundation, Swedish research Council, Crafoord Foundation and The Royal Physiological Society.  

## **Acknowledgements**

We thank the patients and controls who donated samples and the staff of the multidisciplinary HD clinics. 

## **Competing interests**

The authors declare no relevant financial interests.  

## **Correspondence**

Correspondence should be addressed to Maria Björkqvist maria.bjorkqvist@med.lu.se
